# Shwachman–Bodian–Diamond syndrome (SBDS) protein is a direct inhibitor of protein phosphatase 2A (PP2A) activity and overexpressed in acute myeloid leukaemia

**DOI:** 10.1038/s41375-020-0814-0

**Published:** 2020-04-08

**Authors:** Matthew D. Dun, Abdul Mannan, Callum J. Rigby, Stephen Butler, Hamish D. Toop, Dominik Beck, Patrick Connerty, Jonathan Sillar, Richard G. S. Kahl, Ryan J. Duchatel, Zacary Germon, Sam Faulkner, Mengna Chi, David Skerrett-Byrne, Heather C. Murray, Hayley Flanagan, Juhura G. Almazi, Hubert Hondermarck, Brett Nixon, Geoff De Iuliis, Janis Chamberlain, Frank Alvaro, Charles E. de Bock, Jonathan C. Morris, Anoop K. Enjeti, Nicole M. Verrills

**Affiliations:** 1 School of Biomedical Sciences and Pharmacy, Faculty of Health and Medicine, University of Newcastle, Callaghan, NSW Australia; 2 Hunter Medical Research Institute, Cancer Research Program, New Lambton Heights, NSW Australia; 3 School of Chemistry, University of New South Wales, Sydney, NSW Australia; 4 Centre for Health Technologies and the School of Biomedical Engineering, University of Technology Sydney, Sydney, Australia; 5 Lowy Cancer Research Centre and the Prince of Wales Clinical School, University of New South Wales, Sydney, Australia; 6 Children’s Cancer Institute Australia, University of New South Wales, Sydney, NSW Australia; 7 Calvary Mater Hospital, Newcastle, NSW Australia; 8 Reproductive Science Group, Faculty of Science, University of Newcastle, Callaghan, NSW Australia; 9 School of Life Sciences, Faculty of Science, University of Technology Sydney, Ultimo, Australia; 10 John Hunter Children’s Hospital, New Lambton Heights, NSW Australia; 11 NSW Health Pathology, John Hunter Hospital, Lookout Road, New Lambton Heights, NSW Australia; 12 School of Medicine and Public Health, Faculty of Health and Medicine, University of Newcastle, Callaghan, NSW Australia

**Keywords:** Acute myeloid leukaemia, Oncogenes

## To the Editor:

The family of serine/threonine phosphatases (PP2A) frequently shows reduced activity in myeloid leukaemias [1, 2]. This is particularly the case in acute myeloid leukaemias (AML) driven by overexpression or constitutively active c-KIT and FLT3, where PP2A inhibition is required for cell transformation which enhances the activation of oncogenic signalling pathways and promotes anti-apoptotic processes [2–4]. In myeloid malignancies, pharmacological activation of PP2A using Forskolin, the immunosuppressant FTY720 (reviewed in [2]) and the non-immunosuppressive chiral-deoxy analogue of FTY720, AAL(S) has a potential therapeutic benefit [4, 5]. FTY720 is the most well-characterised PP2A-activating compound and phosphorylated by SPHK2 in vivo [6]. FTY720-P acts as a high-affinity agonist for four of the five G protein-coupled sphingosine-1-phosphate receptors (S1PR), causing receptor internalisation and increased activity of the JAK2–PI3Kγ–PKC signalling axis, a common molecular driver of myeloproliferative neoplasms [7]. Phosphorylation has been shown to be dispensable for the anti-leukaemic activity of FTY720 [4, 5]. Furthermore, dose-limiting toxicities are associated with S1PR activation at the anti-neoplastic dose (FTY720-P) [8]. Non-phosphorylated FTY720 binds to the nuclear proto-oncogene SET in leukaemia cells, driving PP2A activity and apoptosis [9]. Non-phosphorylatable analogues of FTY720 also inhibit proliferation and induce apoptosis in several cancer cell lines including AML cell lines and patient samples through the activation of PP2A [5]. However, the molecular targets of these novel chemicals are unknown.

**Fig. 1:**
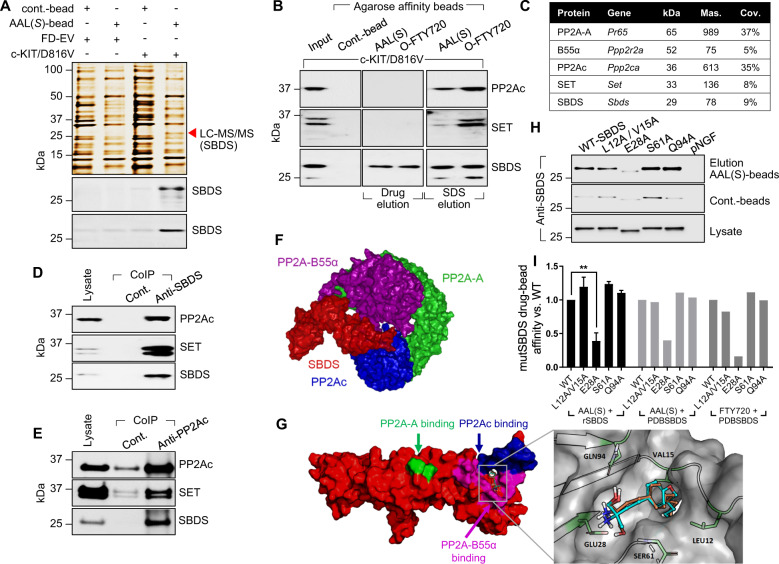
SBDS is target of PP2A-activating drugs and a PP2A-interacting protein. **a** Silver-stained gel of proteins pulled down by AAL(S)-drug affinity beads eluted using excess native drug (250 nM AAL(S)) in factor-dependent (GM-CSF) control myeloid progenitor cells (FD-EV) and c-KIT/D816V cells. The red arrowhead indicates the band excised and subjected to LC-MS/MS. Western blot analysis confirmed SBDS as a target of AAL(S) using both hydrophilic- (top) and hydrophobic- (bottom) AAL(S)-drug affinity beads. **b** Western blot confirmation of SBDS as a target of O-FTY720-beads (hydrophobic-beads) eluted using native drug (250 nM FTY720 or 250 nM AAL(S)) and further eluted using denaturing conditions (SDS). PP2Ac and SET were also eluted using reducing conditions. **c** Proteins identified by LC-MS/MS enriched following SBDS co-immunoprecipitation (CoIP) from c-KIT/D816V cells. **d** Western blot confirmation of SBDS CoIP LC-MS/MS results. **e** Reciprocal CoIP using PP2Ac as bait confirming SBDS–PP2A interactions. **f** Protein–protein docking of SBDS (red) with PP2A subunits Aα (green), B55α (purple) and Cα (blue) predicts SBDS NTD to directly bind to PP2Ac and PP2A–B55α**. g** Docking with SBDS indicates that AAL(S) (orange) and FTY720 (green) form hydrogen bonds (S61, Q94), a salt bridge (E28) and hydrophobic interactions (L12, V15) with the NTD of SBDS, in the region surrounded by predicted binding sites with PP2Ac (blue) and B55α (purple). **h** Site directed mutagenesis induced alanine substitutions; SBDS mutants, L12A/V15A, E28A, S61A and Q94A were overexpressed, purified and used in AAL(S)-drug bead affinity chromatography. SDS-PAGE gel shifting occurred in the E28A mutant to increase gel migration by the binding of additional SDS molecules, without altering the secondary structure. **i** Quantitative assessment of AAL(S) and FTY720 affinity for wild-type (WT) and alanine mutant SBDS using recombinant protein, and in silico alanine mutant PDB SBDS structures.

To determine protein targets of AAL(S) and FTY720 in myeloid progenitor cells driven by constitutively active c-KIT, isogenic growth factor-dependent FDC.P1 myeloid progenitor cell lines expressing either an empty vector (FD-EV) or factor independent c-KIT/D816V [3] were subjected to a chemical proteomics screen. Proteins competitively eluted from AAL(S)- or O-FTY720-agarose affinity beads (Supplementary Fig. S1A) using native drug (250 nM AAL(S) or FTY720) in non-reduced lysates were excised and subjected to LC-MS/MS; identifying the Shwachman–Bodian–Diamond syndrome (SBDS) protein as a direct target (Fig. 1a, b). *SBDS* loss-of-function (LOF) mutations cause the Shwachman–Diamond syndrome (SDS), which leads to bone marrow failure, with SDS patients predisposed to leukaemic transformation, particularly AML [10]. The inhibition of PP2A in myeloid leukaemias is associated with the overexpression of the PP2A inhibitory protein SET [9]; however, using our chemical proteomics approach the catalytic subunit of PP2A, PP2Ac (*PPP2CA)* and SET were only eluted from AAL(S)- or FTY720-beads under denaturing conditions and not following competitive drug elution; albeit considerably less SET was eluted from AAL(S)-beads than from O-FTY720-beads (Fig. 1b). Likewise, SBDS further eluted from the beads using denaturing conditions, suggesting that both monomeric SBDS and PP2Ac macromolecular complexes comprised of SBDS and SET bind to AAL(S) and FTY720.

To independently identify SBDS interacting proteins, SBDS immunoprecipitates from c-KIT/D816V cells were subjected to LC-MS/MS (Supplementary Fig. S1B). Peptides were mapped with high confidence to SBDS, PP2Ac, PP2A structural subunit (PP2A-A), PP2A regulatory subunit B55α (B55α), nucleophosmin (NPM1) and SET (Fig. 1c and Supplementary Fig. S1B). No other PP2A regulatory subunits were identified. These novel and specific interactions were then validated in c-KIT/D816V cells using reciprocal co-immunoprecipitation with both SBDS and PP2Ac antibodies (Fig. 1d, e and Supplementary Fig. S1B).

Potential direct binding of SBDS to PP2A and PP2A-activating drugs AAL(S) and FTY720 was further investigated in silico using all available nuclear magnetic resonance structures of SBDS. This analysis identified an amphipathic binding pocket within the N-terminal domain of SBDS that likely interacts with PP2A–B55α complexes (Fig. 1f). This region corresponded well with binding hot spots identified using the solvent mapping platform FTMap [11] (Supplementary Fig. S1C). We next docked AAL(S) and FTY720 into SBDS, which predicted them to bind Serine 61 (S61) and glutamine (Q94) to form hydrogen bonds, while glutamic acid 28 (E28) formed a salt bridge with the amino alcohol region of AAL(S) and FTY720. Leucine 12 (L12) and valine 15 (V15) were predicted to associate within a small hydrophobic pocket localised in the N-terminal FYSH domain (Fungal, Yhr087wp and Shwachman) (Fig. 1g). Using site directed mutagenesis, amino acids predicted to bind with AAL(S) and FTY720 were modified to alanine and the affinity of AAL(S) to bind SBDS determined biochemically (Supplementary Table S1). Mutagenesis of E28 to alanine (E28A) significantly reduced affinity (−61%, *p* = 0.011) (Fig. 1h, i). Increased affinity for the control beads lacking the amino alcohol moieties of AAL(S) was seen in L12A/V15A and S61A mutants, highlighting a role for the hydrophobic tail of AAL(S) and FTY720 in SBDS binding (Fig. 1h—Cont.-beads). It is interesting to note that the hydrophobic FYSH region of SBDS is predicted to be the site of interaction with PP2Ac and PP2A–B55α subunits (Fig. 1f, g). This interaction may modulate the activity of PP2Ac by blocking substrate phosphopeptides from binding and hence prevent their dephosphorylation. Little to no affinity for either the AAL(S) or control beads was seen using a control protein, recombinant pro-NGF, which has a similar molecular weight as SBDS (27 kDA) and not predicted to bind AAL(S) or FTY720 (Supplementary Fig. S1D). In silico analysis of the binding energies between AAL(S) and FTY720 and the wild-type SBDS (Protein Data Bank (PDB) archive: accession codes SBDS-2L9N [12]) and alanine mutant SBDS proteins also showed that the affinity was greatly reduced for the E28A mutant for both AAL(S) and FTY720 (−60.1% and −84%, respectively) (Fig. 1i). This is due to the removal of the salt bridge interaction between the primary amine head group (on both AAL(S) and FTY720) in the E28A mutant. These data further highlight that AAL(S) and FTY720 can interact with SBDS bound and unbound to PP2Ac/PP2A-A/B55α complexes.

**Fig. 2:**
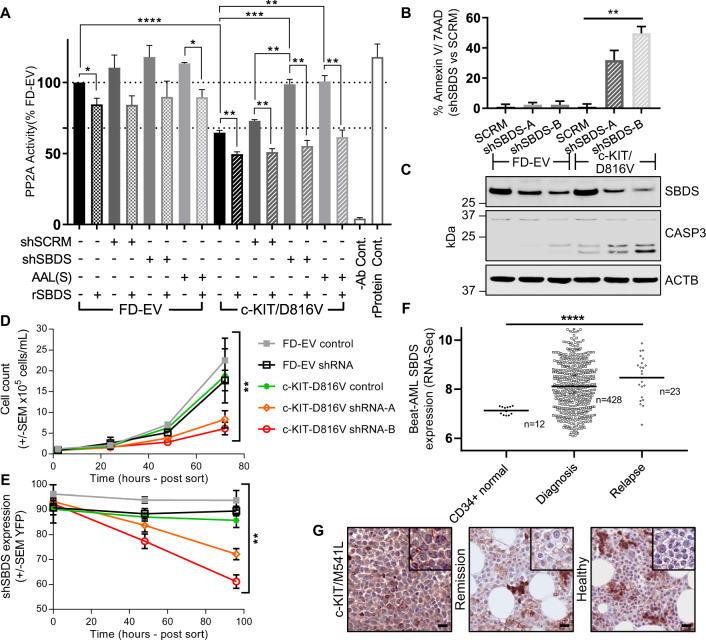
SBDS inhibits PP2A activity and is essential for the survival of c-KIT/D816V mutant cells. **a** PP2A activity was assessed following molecular inhibition of SBDS using SBDS shRNA, or treatment of FD-EV and c-KIT/D816V cells for 12 h with 250 nM AAL(S). FD-EV and c-KIT/D816V cells were transiently transfected with YFP-tagged scrambled shRNA controls or two different YFP-tagged SBDS shRNA constructs for 24 h, and then sorted for populations of cells expressing high YFP. **b** Cells were then stained 24 h post sort with Annexin V, quantitation of dead cells post sort was measured by Annexin V+ and 7AAD+ cells as a percent of scrambled controls. **c** Western blot for caspase 3 following transient SBDS knockdown. **d** c-KIT/D816V cells harbouring the knockdown of SBDS (shSBDS-A, orange; shSBDS-B, red) showed significantly reduced proliferation compared with scrambled shRNA controls (FD-EV, grey; c-KIT/D816V, green) and FD-EV SBDS knockdown (shSBDS, black) determined by the Trypan blue exclusion assay, and **e** reduced YFP+ cells as determined via flow cytometry (**p < 0.01, Two-way ANOVA). **f** *SBDS* RNA-sequencing data from primary AML blast samples from 428 AML patients at diagnosis, 23 patients following AML relapse, and *SBDS* expression in normal bone marrow CD34+ cells from 12 healthy controls. Data extracted from the Beat AML® cohort and were analysed using www.vizome.org. **g** Trephine biopsies from an AML patient harbouring a c-KIT mutation at diagnosis and following successful treatment with standard of care chemotherapies, and a trephine biopsy from a healthy control immunostained for SBDS.

We have previously shown that PP2A activity is reduced in myeloid cells and patients blasts harbouring constitutively active c-KIT/D816V and FLT3/ITD compared with GM-CSF-dependent FD-EV cells and activity is increased using FTY720 and AAL(S) [3–5]. Here, we confirmed this and showed that shRNA-mediated knockdown of SBDS (shSBDS) increased PP2A activity (Fig. 2a), through reduced affinity and co-localisation with PP2A (Supplementary Figs. S2A and S2B, respectively). Notably, the addition of recombinant SBDS to PP2A immunoprecipitates reduced PP2A activity in both the c-KIT/D816V and FD-EV cells (Fig. 2a).

*Sbds* LOF mutations in cells of the myeloid lineage drives apoptosis in myelocytes and their respective downstream progeny, while loss of *Sbds* is well tolerated by cycling haematopoietic progenitor cells [10]. Transient knockdown of SBDS in constitutively active c-KIT/D816V myeloid cells increased cleaved caspase 3 and apoptosis compared with their scrambled shRNA controls (SCRM) (Fig. 2b, c and Supplementary Fig. S2C), while it decreased cell proliferation (Fig. 2d). However, factor-dependent FD-EV myeloid cells showed no reduction in viability (Fig. 2d) or proliferation compared with their controls. This was further reflected by a reduction in the percentage of cells expressing the SBDS knockdown over time only in the c-KIT/D816V cells (Fig. 2e). Taken together, these data showed that the inhibition of SBDS increased PP2A activity, inhibited proliferation and induced cell death in myeloid cells harbouring constitutively active c-KIT/D816V. Whether LOF of *SBDS* in myleocytes of SDS patients drives overactivation of PP2A and promotes apoptotic process remains to be determined, but if true would suggest that therapies that reduce PP2A activity may help to reduce dysfunctional myeloblast differentiation commonly seen in SDS patients.

To determine the clinical relevance of SBDS expression in AML, we assessed *SBDS* mRNA expression in normal bone marrow CD34+ cells from healthy subjects (*n* = 12), and compared expression with AML patient blasts at diagnosis (*n* = 428) and AML patient blasts at relapse (*n* = 23) using the publicly available Beat AML data viewer (www.vizome.org) [13, 14]. These analyses showed that at diagnosis, AML patients expressed significantly more *SBDS* mRNA than healthy controls (Mann–Whitney test *p* = 0.0002), while patients that relapsed after chemotherapy expressed significantly more *SBDS* mRNA than both healthy controls and patients at diagnosis (Kruskal–Wallis test *p* = 0.0004) (Fig. 2f), highlighting SBDS as a novel drug target. To determine the potential of SBDS protein as a drug target we assessed SBDS protein expression via immunohistochemistry using bone marrow trephine biopsies from a core binding factor-AML patient (Inv(16)(p13.1;q22) also harbouring a c-KIT mutation (M541L). This analysis showed high SBDS expression at diagnosis, whereas no SBDS protein expression was seen in their matched remission sample following standard of care treatment, or in normal myeloblasts from a healthy control (Fig. 2g). Although the mechanism underpinning the increased SBDS mRNA and protein expression in AML blasts both at diagnosis and relapse are not known, these data go some way to describe why AML patients myeloblasts show reduced PP2A activity compared with normal myeloblasts [1, 4].

Our study provides a novel mechanism of PP2A inhibition in myeloid progenitor cells harbouring mutant c-KIT. SBDS is a target of FTY720 and AAL(S), with SBDS expression essential for the factor independent survival of these mutant c-KIT cells. Significantly higher *SBDS* expression is seen in AML patients at diagnosis, exacerbated at relapse, highlighting the need for future investigations focused on the role SBDS plays in leukemogenesis and future drug targeting, particularly in the relapse setting [15].

## Supplementary information

Supplementary Information

Supplementary Figures
