# Supplementary material for: Shwachman–Bodian–Diamond syndrome (SBDS) protein is a direct inhibitor of protein phosphatase 2A (PP2A) activity and overexpressed in acute myeloid leukaemia
Source: Leukemia. 2020 Apr 8;34(12):3393–7. doi: 10.1038/s41375-020-0814-0 (PMC7685970; doi:10.1038/s41375-020-0814-0)
Supplement: Supplementary file 1 — Supplementary Information [file 41375_2020_814_MOESM1_ESM.docx]

**Supplementary Information**

**Supplementary Methods**

**Reagents**

Unless specified otherwise, all reagents were obtained from Sigma-Aldrich (St. Louis, Mo, USA) or Thermo Fisher Scientific (Waltham, MA, USA) and were of research grade. Modified trypsin was from Promega (Madison, WI, USA). Poros R2 and Poros Oligo R3 reverse-phase material were from Applied Biosystems (Forster City, CA, USA). GELoader tips were from Eppendorf (Hamburg, Germany). The 3M EmporeTM C8 disk was from 3M Bioanalytical Technologies (St. Paul, MN, USA). All solutions were made with ultrapure Sigma water (Sigma-Aldrich). Fingolimod (FTY720) was purchased from Cayman Chemicals (Ann Arbor, MI, USA). AAL(S) was synthesized as described. ^1^

**Cell lines, retroviral infection, and survival assays**

The FDC.P1 growth factor–dependent mouse myeloid progenitor cell line ^2^ stably expressing an empty vector (EV) or the imatinib-resistant human c-KIT/D816V were used as previously described. ^3^ Stable and transient knockdown of SBDS was achieved in FDC.P1 cells using shRNA targeting the mouse *Sbds* gene or a non-specific control sequence (shSCRM) in the pFSYci vector with a YFP reporter gene under the control of a bicistronic promoter (a kind gift from Dr. Daniel Link, Washington University School of Medicine, St Louis, USA and prepared as described ^4^). Transient molecular inhibition was achieved by lentiviral transduction of FD-EV and c-KIT/D816V cells with shRNA viral supernatant for 24 hours. Cells were then sorted on a FACSAria (BD Biosciences, San Jose, CA, USA) into populations expressing high or low YFP, as a proxy marker for relative SBDS knockdown. Annexin V staining was performed 24 hours post sorting. ^5^ Cell growth and YFP expression was monitored for 6 days by Trypan blue viability staining and flow cytometry, respectively. In addition, stable cell lines were selected by sorting populations of YPF+ cells. ^4^

**Synthesis of click agarose beads for affinity chromatography**

Structure activity relationship data confirmed that the amino-alcohol head group was essential for biological activity, ^1^ therefore affinity beads were attached to the hydrophobic tail as this part of the molecule was shown to be less important for the cytotoxicity and PP2A activating capacity of the analogues. Finn and co-workers developed click reactions utilized to couple a molecule of interest to an agarose bead for affinity chromatography. ^6^ Thus, using our recently reported modular synthesis of AAL(S) analogues, we synthesized an analogue of AAL(S) with a terminal acetylene on the hydrophobic tail from the previously reported bis-lactim ether ^7^ shown in **Supplementary** **Figure S1A**. Before embarking on the probe synthesis a test substrate was synthesised to see if functionalisation at the end of the hydrophobic tail would affect activation of PP2A. Terminal acetylene AAL(S) analogue was subjected to click reaction conditions (CuI, TBTA, DIPEA, DMF) in the presence of t-butyl (2-(2-(2-azidoethoxy)ethoxy)ethyl)carbamate which afforded triazole with a 53 % yield.

A similar protocol was applied to the synthesis of an FTY720 analogue where we incorporated an oxygen into the hydrophobic tail of FTY720 (**Supplementary Figure S1A**). O-FTY720 is only slightly less cytotoxic in c-KIT/D816V cells (IC_50_ = 5.7 μM) as FTY720 (IC50 = 3.6 μM) and AAL(S) (IC50 = 3.7 μM). ^1^ The AAL(S) and O-FTY720 affinity chromatography substrates were attached to a terminal azide solid support using the method developed by Finn to afford AAL(S) and O-FTY720 affinity chromatography probes in 16 and 45 % yields respectively. A negative control bead was also synthesised in a similar manner **Supplementary** **Figure S1A – Control**).

**PP2A activating drug (PAD) affinity chromatography**

Cells were lysed in PP2A activity assay buffer ^8^ and 500 µg of protein lysate was incubated with 50 µL of control beads at 4 °C for 2 hours before incubation with AAL(S) or O-FTY720 beads at 4 ^°^C overnight. Control and active beads were washed 3 times in sterile Tris-buffered saline (TBS), 0.01% Tween 20 v/v for 5 minutes at room temperature. Bound proteins were first eluted using a competitive elution strategy employing 250 nM native AAL(S) or FTY720 for 10 minutes at room temperature. The remaining bound proteins were subjected to reducing conditions by boiling in 1× NuPAGE® LDS Sample Buffer (Invitrogen, Carlsbad, CA, USA) containing 2% β-mercaptoethanol v/v for 5 minutes. Proteins were separated, silver stained ^9^ and proteins that bound uniquely to active beads from c-KIT/D816V cell lysates were excised and subjected to liquid chromatography tandem mass spectrometry (LC-MS/MS) as previously described. ^10^ MS results were searched using Mascot 2.2 using Uniprot_mouse database and protein reported if Mascot score was ≥67.

AAL(S) drug-bead affinity purification was performed on bacterially expressed and purified recombinant wild type and mutant SBDS to confirm the predicted affinity of AAL(S) and FTY720 with Serine 61 (S61), glutamine (Q94), glutamic acid 28 (E28), leucine 12 (L12) and valine 15 (V15) (see **SBDS bacterial expression and site directed mutagenesis**, below) (**Figure 1H, and I**). To confirm interactions, 50 ng of recombinant wild type and mutant SBDS were mixed with 105 µl AAL(S) beads in TBS (pH 7.35) for 1 hour mixing at 4 °C. Following, unbound SBDS was cleared and beads washed three times in TBS. For competitive elution 250 nM AAL(S) in 200 µL of TBS was incubated at 25 °C for 10 minutes at 500 rpm in thermomixer. Tubes were immediately transferred to ice, after gentle pelleting, supernatant was collected. To reduce any remaining interactions, 1 x SDS-PAGE loading buffer was added to each bead pellet and heated for 5 minutes at 100 °C. Each elution was loaded into SDS-PAGE, transferred via Western blotting (as described below) and probed for anti-SBDS and anti-proNGF. Recombinant proNGF (50 ng) was used as per SBDS to act as a negative control (expressed and described ^11^).

**Immunohistochemistry of bone marrow samples**

Studies were approved by the Hunter New England Area Health Human Ethics Committee. Informed consent was obtained from the participants according to institutional guidelines and ethical standards of Helsinki declaration. Matched human bone marrow aspirates were obtained from a core binding factor-AML patient (Inv(16)(p13.1;q22), harbouring a c-KIT mutation (M541L) at diagnosis and then following relapse from standard of care chemotherapies, treated at the Calvary Mater Newcastle (CMN) Hospital Australia. Diagnosis was confirmed using cytomorphology, cytogenetics, and flow cytometry according to the World Health Organization (WHO) classification of the myeloid neoplasms. ^12^ Cytogenetic risk classification categories were defined according to the Medical Research Council schema. c-KIT status was assessed using high-resolution melt analysis and direct sequencing from Ficoll-purified mononuclear cells as described. ^5^ Immunohistochemical evaluation of SBDS expression was performed using the automated tissue staining facility at the Berghofer Queensland Institute of Medical Research.

*SBDS* RNA-Seq data was downloaded and viewed using Beat AML data viewer (www.vizome.org) and consisted of RNA-sequencing data from primary specimens from 463 AML patients with detailed clinical annotations, including diagnostic information, treatments, responses and outcomes treated on the Beat-AML Trial ^13, 14^ on the 10^th^ of November 2019. Differences in *SBDS* mRNA expression was determined between groups using Mann-Whitney tests and across groups using Kruskal-Wallis tests.

**Western blotting, co-immunoprecipitation, PP2A activity assay**

Whole cell lysates for Western blot analysis were prepared using ice cold RIPA buffer ^15^ containing 5 mM Na_3_VO_4_, protease inhibitors (Complete; Roche, Basel, Switzerland) and PhosSTOP (Roche). Cells were probe-tip sonicated for 2 × 20 seconds on ice and mixed for 30 minutes at 4 ^°^C. Western blot analysis was performed using anti-SBDS (sc-271350), anti-PP2A-B56α (sc-271151), anti-SET (sc-133138), (Santa Cruz Biotechnology, Dallas, TX, USA), anit-NPM1 (3542) (Cell Signaling), anti-SBDS (NIC3) (Genetex, Irvine, CA, USA), in-house anti-PP2Ac ^16^ and commercial anti-PP2Ac (05-421), anti-PP2A-B55α (05-592), anti-PP2A-A (07-250), anti-Caspase 3 (AB1899) (Millipore, Burlington, MA, USA), and anti-β-actin (Sigma-Aldrich). Secondary, horseradish peroxidase (HRP) conjugated antibodies (1662408) (BioRad, Hercules, CA, USA), and secondary native anti-mouse-HRP (18-8817-30) (Rockland Immunochemicals, Limerick, PA, USA). Bands were visualised using a cooled charge coupled device camera (ImageQuant LAS-4000; GE Healthcare, Chicago, IL, USA). ^10^ SBDS and PP2A interacting proteins were identified by co-immunoprecipitation coupled to LC-MS/MS as described. ^9, 10^ PP2A activity assay was performed as described, using anti-PP2Ac antibody (1D6) (Millipore). ^5, 8^

**Immunolocalisation**

Cells were washed in phosphate-buffered saline (PBS) twice and diluted to 5 × 10^5^ cells/ml. Slides were coated with foetal calf serum (FCS) and 100 μL of cell suspension was centrifuged onto each glass slide at 500 × g for 5 minutes. Cells were fixed in 3.7% paraformaldehyde/PBS for 10 minutes, washed 3 times with PBS, permeabilised with 0.1% Triton X-100 for 3 minutes and blocked in 10% FCS/PBS at room temperature for 20 minutes. Primary incubation was undertaken with 1:100 dilution of primary antibody (SBDS – Genetex, PP2Ac – in house) at 4 °C overnight. Slides were subjected to 3 × 5 minute washes with PBS and incubated in a 1:500 dilution of the appropriate Alexa Fluor conjugated secondary antibody (BD Biosciences, Franklin Lakes, NJ, USA) at room temperature for 45 minutes, then washed and mounted in ProLong Gold anti-fade with DAPI (Life Technologies, Carlsbad, CA, USA), and imaged using a LSM510 laser scanning confocal microscope (Carl Zeiss, Oberkochen, Germany). ^10^

**Molecular modelling**

All proteins used were prepared by the addition of protons, protonation at biological pH, and restrained minimisation using an optimised potentials for liquid simulations (OPLS3) force field. Protein structures were taken from the Research Collaboratory for Structural Bioinformatics Protein Data Bank archive (RCSB PDB): accession codes 3DW8 for PP2A (Protein Phosphatase 2A Holoenzyme with B55α) ^17^ 2L9N for SBDS ^18^ and 2E50 for SET ^19^. Each of the 20 available conformers of SBDS was prepared separately, and assigned a number, 2L9N_1-20, as per the original designation in the PDB file. ^18^ Solvent mapping was conducted using the FTMap web server, and averaged across all 20 SBDS conformers ^20^ (**Supplementary Figure S1C**). Protein-protein docking was conducted using the Cluspro web server. ^21-23^ Results were analysed with ‘balanced’ coefficients, and ranking was based on cluster size, model scores, and comparison with results from solvent mapping. Automated binding site detection was conducted using Schrodinger’s SiteMap, implemented through Maestro 11.0, both with and without the recommended pre-set values for shallow binding sites. ^24, 25^ The top five ranked binding sites were retained for consideration. All ligands were prepared for use by protonation at biological pH and minimisation with an OPLS3 force field. Ligand-protein docking was conducted using Extra Precision Glide, implemented through Schrodinger’s Maestro 11.0. ^26^

**SBDS bacterial expression and site directed mutagenesis**

The bacterial recombinant protein expression plasmid pGEX-4T1 (GE Life Science, PA, USA) was used to induce expression of recombinant human SBDS (a generous gift from Professor Akiko Shimamura, Pediatric Hematology-Oncology, Boston Children's Hospital, Boston, MA, USA; Dana Farber Cancer Institute, Boston, MA, USA; Department of Pediatrics, Harvard Medical School, Boston, MA, USA used as described ^27^). Briefly, endonuclease *EcoRI* (GE Life Sciences, Pittsburgh, PA, USA) was used with the SBDS gene (SBDS_HUMAN, UniProt: Q9Y3A5) containing complementary flanking regions and ligated using T4 DNA Ligase (New England Biolabs, Ipswich, MA, USA). One Shot™ BL21(DE3) Escherichia Coli (E.Coli) (Thermo Fisher, Waltham, MA, USA) were transfected with 10 ng of pGEX-4T1 subcloned with the SBDS-GST fusion protein using standard heat shock method transfection protocol. ^28^ Transfected BL21 (De3) E-Coli bacterial cells grown at 37°C (1hr) in liquid broth were plated onto Luria-Bertani (LB) agar (1.2% w/v bacteriological agar) (Fisher Scientific, Australia) containing antibiotics (Ampicillin 100 μg/mL) for selection. After 24hr of growth at 37°C healthy and discrete colonies were collected to prepare glycerol stocks of transfected competent BL21 (DE3) cells. Vector DNA was isolated using ISOLATE II Plasmid Mini Kit (Biolin Meridian Bioscience, NSW, Australia), and sent sequencing to Australian Genomic Research Facility (AGRF, NSW Australia), then used for site directed mutagenesis. Primers (**Supplementary Table S1**) were used for site directed mutagenesis using QuikChange II Site-Directed Mutagenesis Kit (Integrated sciences, NSW, Australia) as per manufacturer’s instructions. Following the isolation of vector DNA, DNA was sent for sequencing again to AGRF. Mutations were confirmed by aligning sequencing data with the WT human recombinant SBDS gene using Geneious software (V 5.0.4).

After site directed mutagenesis, 10 ng of pGEX-4T1 containing mutant and wild type rSBDS-GST fusion proteins were used to transfect one Shot™ BL21(DE3) Escherichia Coli (E.Coli) (Thermo Fisher, Waltham, MA, USA) using standard heat shock method transfection protocol. Single colony of transfected BL21 (De3) E-Coli bacterial cells growing in ampicillin (100 µM) containing LB agarose were picked, and added to 10mL of ampicillin containing LB for overnight (O/N) culture in an orbital shaker at 250 rpm and 37 °C. O/N culture was added to 180 mL of ampicillin containing LB and OD density at 600 (OD600), monitored after every hour. At OD600 of ~5, the LacZ operon transcription initiation lactose metabolite Isopropyl β-D-1-thiogalactopyranoside (IPTG) was used to trigger the expression of recombinant SBDS- GST tagged fusion protein (rSBDS) at a concentration of 200 µM. After 3 hours of growth bacterial growth at RT, cell pellets were washed twice with TBS and stored at -80 °C. Cell lysates were prepared in TBS Triton 100X (v/v 1%) pH 7.4, and lysates were clarified by centrifugation at 4000 rpm for 30 minutes. GE SpinTrap™ glutathione bound 4B Sepharose columns (GE, Pittsburgh, PA, USA) were used to bind recombinant SBDS-GST fusion protein. Thrombin (8U) was used to cleave GST bound rSBDS and removed using HiTrap® Benzamidine Fast Flow 5mL (GE, Pittsburgh, PA, USA). Purified rSBDS was stored at -80 °C

**Supplementary Table S1. Primer sequences used to perform site directed mutagenesis on wild type *SBDS* gene.**

| Primer | Sequence | Tm (°C) |
| --- | --- | --- |
| SBDS L12A/V15A For | 5'-CCACCAACCAGATCCGCGCAACCAATGCGGCCGTGGTACGGATGAAGCGTGCCG-3' | 68 |
| SBDS L12A/V15A Rev | 5'-CGGCACGCTTCATCCGTACCACGGCCGCATTGGTTGCGCGGATCTGGTTGGTGG-3' | 68 |
| SBDS E28A For | 5'-GCGTGCCGGGAAGCGCTTCGCAATCGCCTGCTACAAAAACAAGG-3' | 68 |
| SBDS E28A Rev | 5'-CCTTGTTTTTGTAGCAGGCGATTGCGAAGCGCTTCCCGGCACGC-3' | 68 |
| SBDS S61A For | 5'-CCCACTCAGTGTTTGTAAATGTTGCGAAAGGTCAGGTTGCCAAAAAGG-3' | 68 |
| SBDS S61A Rev | 5'-CCTTTTTGGCAACCTGACCTTTCGCAACATTTACAAACACTGAGTGGG-3' | 68 |
| SBDS Q94A For | 5'GCAGATTTTGACTAAAGGAGAAGTTGCAGTATCAGATAAAGAAAGACACACACAACTGG-3' | 68 |
| SBDS Q94A Rev | 5'CCAGTTGTGTGTGTCTTTCTTTATCTGATACTGCAACTTCTCCTTTAGTCAAAATCTGC-3' | 68 |

Tm, melting temperature. For, forward primer. Rev, reverse primer.

**Supplementary Figure Captions**

**Supplementary Figure S1:** **SBDS is a PP2A interacting protein.** **(A)** Structure of immobilized diaminodipropylamine drug beads. Control-beads lack the functional group of the PP2A activating drugs. **(B)** Silver-stained SDS-PAGE and corresponding Western blot confirmation of c-KIT/D816V SBDS co-immunoprecipitation, proteins eluted from control beads or SBDS CoIP using 100 mM glycine, pH 2.5. Bands eluted following SBDS pulldown were excised and subjected to LC-MS/MS. **(C)** Predicted amino acids facilitating SBDS and PP2Aα/Bα/Cα integrations. Binding hot spots identified using the solvent mapping platform FTMap. ^20^ **(D)** AAL(S) drug bead affinity with recombinant pro-NGF.

**Supplementary Figure S2**. **SBDS knockdown or treatment of c-KIT/D816V cells with AAL(S) alters PP2A activity and survival of myeloid progenitor cells harbouring mutant c-KIT/D816V. (A)** Western-blot assessment of SBDS association with PP2Ac immunoprecipitated complexes in cells with and without shRNA mediated knockdown of SBDS, and with (+) and without (-) the addition of recombinant SBDS or 2.5 µM AAL(S). **(B)** Co-localisation (yellow) of SBDS (red) and PP2Ac (green) was assessed by immunofluorescence analyses in FD-EV and c-KIT/D816V cells following 90 minutes treatment with 2.5 µM AAL(S). Blue, DAPI maker of DNA (scale bars = 40 µm, **p*<0.05 determined by 2-tail Students t-test). **(C)** FD-EV and c-KIT/D816V cells were transiently transfected with YFP-tagged scrambled DNA shRNA control, or two different YFP-tagged SBDS shRNA constructs for 24 hours and then sorted for populations of cells expressing high YFP. Cells were then stained 24 hours post sort with Annexin V. Quantitation of dead cells post sort measured by Annexin V+ and 7AAD+ cells is shown in **Figure 2B**.

**Supplementary References**

1. Toop HD, Dun MD, Ross BK, Flanagan HM, Verrills NM, Morris JC. Development of novel PP2A activators for use in the treatment of acute myeloid leukaemia. *Org Biomol Chem* 2016 May 18; **14**(20)**:** 4605-4616.

2. Dexter TM, Garland J, Scott D, Scolnick E, Metcalf D. Growth of factor-dependent hemopoietic precursor cell lines. *J Exp Med* 1980 Oct 01; **152**(4)**:** 1036-1047.

3. Frost MJ, Ferrao PT, Hughes TP, Ashman LK. Juxtamembrane mutant V560GKit is more sensitive to Imatinib (STI571) compared with wild-type c-kit whereas the kinase domain mutant D816VKit is resistant. *Mol Cancer Ther* 2002 Oct; **1**(12)**:** 1115-1124.

4. Rawls AS, Gregory AD, Woloszynek JR, Liu F, Link DC. Lentiviral-mediated RNAi inhibition of Sbds in murine hematopoietic progenitors impairs their hematopoietic potential. *Blood* 2007 Oct 01; **110**(7)**:** 2414-2422.

5. Roberts KG, Smith AM, McDougall F, Carpenter H, Horan M, Neviani P*, et al.* Essential requirement for PP2A inhibition by the oncogenic receptor c-KIT suggests PP2A reactivation as a strategy to treat c-KIT+ cancers. *Cancer Res* 2010 Jul 01; **70**(13)**:** 5438-5447.

6. Punna S, Kaltgrad E, Finn MG. "Clickable" agarose for affinity chromatography. *Bioconjug Chem* 2005 Nov-Dec; **16**(6)**:** 1536-1541.

7. Schollkopf U, Groth U. Asymmetric-Synthesis Via Heterocyclic Intermediates .9. Enantioselective Synthesis of (R)-Alpha-Vinylamino Acids. *Angew Chem Int Edit* 1981; **20**(11)**:** 977-978.

8. Smith AM, Dun MD, Lee EM, Harrison C, Kahl R, Flanagan H*, et al.* Activation of protein phosphatase 2A in FLT3+ acute myeloid leukemia cells enhances the cytotoxicity of FLT3 tyrosine kinase inhibitors. *Oncotarget* 2016 Jul 26; **7**(30)**:** 47465-47478.

9. Dun MD, Anderson AL, Bromfield EG, Asquith KL, Emmett B, McLaughlin EA*, et al.* Investigation of the expression and functional significance of the novel mouse sperm protein, a disintegrin and metalloprotease with thrombospondin type 1 motifs number 10 (ADAMTS10). *Int J Androl* 2012 Aug; **35**(4)**:** 572-589.

10. Dun MD, Smith ND, Baker MA, Lin M, Aitken RJ, Nixon B. The chaperonin containing TCP1 complex (CCT/TRiC) is involved in mediating sperm-oocyte interaction. *J Biol Chem* 2011 Oct 21; **286**(42)**:** 36875-36887.

11. Demont Y, Corbet C, Page A, Ataman-Onal Y, Choquet-Kastylevsky G, Fliniaux I*, et al.* Pro-nerve growth factor induces autocrine stimulation of breast cancer cell invasion through tropomyosin-related kinase A (TrkA) and sortilin protein. *J Biol Chem* 2012 Jan 13; **287**(3)**:** 1923-1931.

12. Vardiman JW, Thiele J, Arber DA, Brunning RD, Borowitz MJ, Porwit A*, et al.* The 2008 revision of the World Health Organization (WHO) classification of myeloid neoplasms and acute leukemia: rationale and important changes. *Blood* 2009 Jul 30; **114**(5)**:** 937-951.

13. Tyner JW, Tognon CE, Bottomly D, Wilmot B, Kurtz SE, Savage SL*, et al.* Functional genomic landscape of acute myeloid leukaemia. *Nature* 2018 Oct; **562**(7728)**:** 526-531.

14. Tang Z, Kang B, Li C, Chen T, Zhang Z. GEPIA2: an enhanced web server for large-scale expression profiling and interactive analysis. *Nucleic Acids Res* 2019 Jul 2; **47**(W1)**:** W556-W560.

15. Alcaraz C, De Diego M, Pastor MJ, Escribano JM. Comparison of a radioimmunoprecipitation assay to immunoblotting and ELISA for detection of antibody to African swine fever virus. *J Vet Diagn Invest* 1990 Jul; **2**(3)**:** 191-196.

16. Sim AT, Collins E, Mudge LM, Rostas JA. Developmental regulation of protein phosphatase types 1 and 2A in post-hatch chicken brain. *Neurochem Res* 1998 Apr; **23**(4)**:** 487-491.

17. Cho US, Xu W. Crystal structure of a protein phosphatase 2A heterotrimeric holoenzyme. *Nature* 2007 Jan 04; **445**(7123)**:** 53-57.

18. Hilcenko C, Freund SMV, Warren AJ. Structure of the Human Shwachman-Bodian-Diamond Syndrome (SBDS) Protein. RSCB PDB; 2011.

19. Muto S, Senda M, Akai Y, Sato L, Suzuki T, Nagai R*, et al.* Relationship between the structure of SET/TAF-Ibeta/INHAT and its histone chaperone activity. *Proc Natl Acad Sci U S A* 2007 Mar 13; **104**(11)**:** 4285-4290.

20. Kozakov D, Grove LE, Hall DR, Bohnuud T, Mottarella SE, Luo L*, et al.* The FTMap family of web servers for determining and characterizing ligand-binding hot spots of proteins. *Nat Protocols* 2015 05//print; **10**(5)**:** 733-755.

21. Comeau SR, Gatchell DW, Vajda S, Camacho CJ. ClusPro: an automated docking and discrimination method for the prediction of protein complexes. *Bioinformatics* 2004; **20**(1)**:** 45-50.

22. Kozakov D, Beglov D, Bohnuud T, Mottarella SE, Xia B, Hall DR*, et al.* How good is automated protein docking? *Proteins: Structure, Function, and Bioinformatics* 2013; **81**(12)**:** 2159-2166.

23. Kozakov D, Hall DR, Xia B, Porter KA, Padhorny D, Yueh C*, et al.* The ClusPro web server for protein-protein docking. *Nat Protocols* 2017 02//print; **12**(2)**:** 255-278.

24. Halgren TA. New Method for Fast and Accurate Binding-site Identification and Analysis. *Chem Biol Drug Des* 2007; **69:** 146-148.

25. Halgren TA. Identifying and Characterizing Binding Sites and Assessing Druggability. *J Chem Inf Model* 2009; **49:** 377-389.

26. Friesner RA, Banks JL, Murphy RB, Halgren TA, Klicic JJ, Mainz DT*, et al.* Glide: A new approach for rapid, accurate docking and scoring. 1. Method and assessment of docking accuracy. *J Med Chem* 2004; **47:** 1739-1749.

27. Gluck L, Alazay L, Ratel D, Puget S, Wion D. Quality control of plasmid preparations. *Nat Biotechnol* 2001 Aug; **19**(8)**:** 715.

28. Froger A, Hall JE. Transformation of plasmid DNA into E. coli using the heat shock method. *J Vis Exp* 2007; (6)**:** 253.
