# Supplementary material for: Shwachman–Bodian–Diamond syndrome (SBDS) protein is a direct inhibitor of protein phosphatase 2A (PP2A) activity and overexpressed in acute myeloid leukaemia
Source: Leukemia. 2020 Apr 8;34(12):3393–7. doi: 10.1038/s41375-020-0814-0 (PMC7685970; doi:10.1038/s41375-020-0814-0)
Supplement: Supplementary file 2 — Supplementary Figures [file 41375_2020_814_MOESM2_ESM.pptx]

## Slide 1
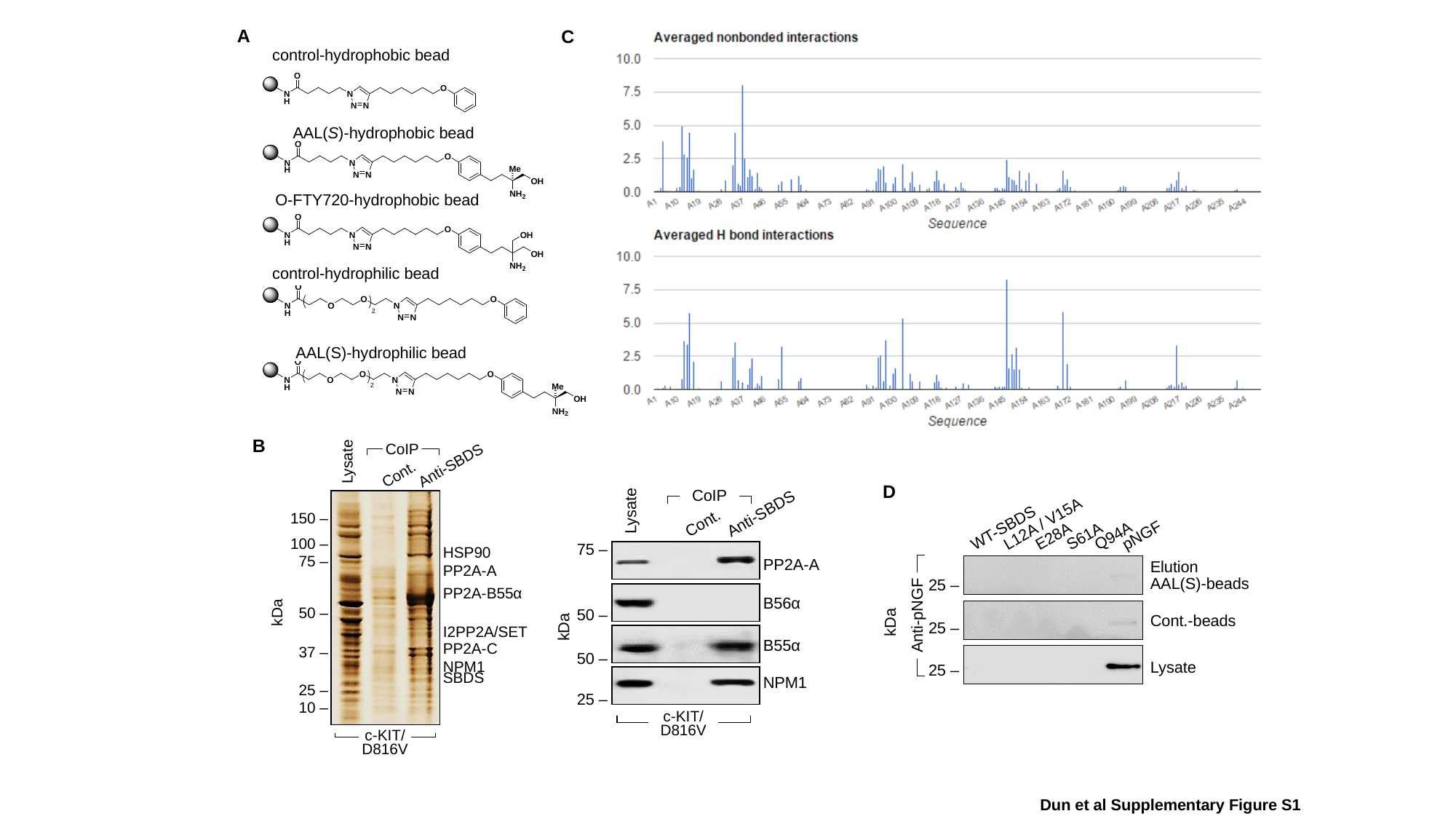

C
A
control-hydrophobic bead
O-FTY720-hydrophobic bead
control-hydrophilic bead
AAL(S)-hydrophilic bead
AAL(S)-hydrophobic bead
Lysate
B
CoIP
Cont.
Anti-SBDS
CoIP
Lysate
Cont.
Anti-SBDS
75 –
PP2A-A
B56α
50 –
kDa
B55α
50 –
NPM1
25 –
c-KIT/
D816V
D
WT-SBDS
L12A / V15A
E28A
S61A
Q94A
pNGF
Elution AAL(S)-beads
Anti-pNGF
Cont.-beads
Lysate
25 –
25 –
25 –
150 –
100 –
75 –
50 –
37 –
25 –
10 –
kDa
HSP90
PP2A-A
PP2A-B55α
kDa
I2PP2A/SET
PP2A-C
NPM1
SBDS
c-KIT/
D816V
Dun et al Supplementary Figure S1

## Slide 2
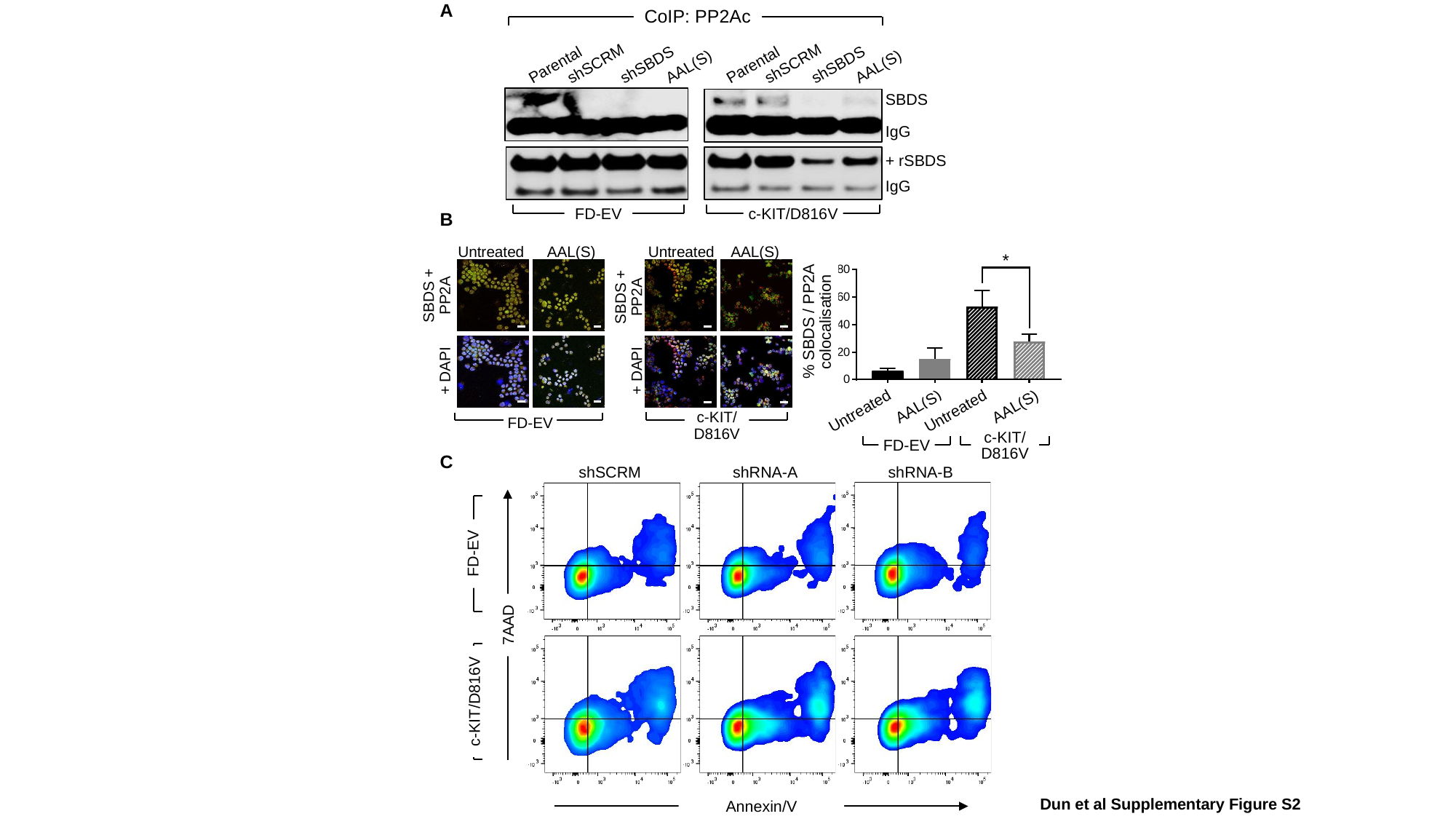

A
CoIP: PP2Ac
Parental
shSCRM
Parental
shSCRM
shSBDS
AAL(S)
shSBDS
AAL(S)
SBDS
IgG
+ rSBDS
IgG
FD-EV
c-KIT/D816V
B
Untreated
AAL(S)
Untreated
AAL(S)
SBDS +
PP2A
SBDS +
PP2A
% SBDS / PP2A colocalisation
+ DAPI
+ DAPI
Untreated
AAL(S)
Untreated
AAL(S)
c-KIT/
D816V
FD-EV
c-KIT/
D816V
FD-EV
C
shSCRM
shRNA-A
shRNA-B
7AAD
FD-EV
c-KIT/D816V
Annexin/V
Dun et al Supplementary Figure S2
